# Editorial: Dietary diversity indicators: cultural preferences and health outcomes

**DOI:** 10.3389/fnut.2024.1433735

**Published:** 2024-06-19

**Authors:** Minatsu Kobayashi, Frank Thielecke

**Affiliations:** ^1^Department of Food Science, Otsuma Women's University, Tokyo, Japan; ^2^Swiss Distance University of Applied Sciences, Brig, Switzerland

**Keywords:** dietary diversity, nutritional status, pregnancy, birth outcomes, public health interventions, nutrition in the elderly, community partnerships

Dietary diversity, which refers to the total number of different foods consumed per day or per week and their association with health outcomes, can be used to improve nutritional outcomes. Even individuals who are unsure about what constitutes a healthy diet can enhance their nutritional status by increasing dietary diversity. The World Health Organization (WHO) and other health organizations recommend consuming a diverse range of food groups to prevent nutrient deficiencies and chronic illnesses (1). Dietary guidelines in many developed countries recommend consuming a variety of foods (2). In Japan, the dietary guidelines established in 1985 advised a daily intake of 30 different foods to prevent nutritional deficiencies and diseases. The current guidelines no longer specify the recommended number of foods per day but emphasize a “well-balanced intake based on staple foods, main dishes, and side dishes,” and the “combination of diverse foods” (3). Previous epidemiological studies suggest that dietary diversity is closely associated with reduced mortality and lower incidence of diseases (4–10). Designing simple indicators that reflect nutritional sufficiency has become a priority for many in the field of public health nutrition.

Focusing for example on the most vulnerable population epidemiological studies on the association between dietary diversity during pregnancy and birth outcomes indicate that maternal nutritional status is crucial for fetal growth and development. Pregnant women need to consume adequate amounts of macronutrients such as carbohydrates, fats, and proteins, as well as micronutrients including iron, zinc, and various vitamins. These nutrients are essential for fetal growth, brain development, and skeletal development. A nutrient-rich diet ensures adequate birth weight and significantly reduces the risk of low birth weight. Adequate nutrition is particularly important because low birth weight is associated with neonatal health problems and future developmental delays.

The adequate intake of folic acid early in pregnancy significantly reduces the risk of neural tube defects. For this reason, folic acid supplementation is recommended in many countries for women planning pregnancy, even before conception. By consuming a variety of foods rich in B vitamins, a mother can simultaneously receive folic acid and other important nutrients that promote the healthy development of the fetus.

High dietary diversity provides balanced nutrition, which helps maintain proper blood glucose control and blood pressure, reducing the risk of gestational diabetes and gestational hypertension (11). Healthy eating habits also contribute to the prevention of overweight and obesity during pregnancy and reduce long-term health risks (12).

Mothers with good nutritional status have been shown to be more likely to maintain good mental health during pregnancy (13). Malnutrition may increase the risk of anxiety and depression, but nutrient-rich diets have been reported to reduce these symptoms (14).

Four interesting papers were submitted to our Research Topics, each presenting significant findings:

A study in Nekemte town, western Ethiopia, examined the association between adequate dietary diversity and related factors among pregnant women. Factors identified as associated with adequate dietary diversity included the wealth index, maternal care, women's occupation, household food insecurity, gestational age, and stable meal frequency. The study suggests that multi-sectoral collaboration is essential to increase dietary diversity among pregnant women by promoting women's employment and strengthening sustainable income-generating activities.

In Bahir Dar, Ethiopia, the prevalence and determinants of the double burden of malnutrition among mothers and their children were assessed at the household level. Significant associations were found with the wealth index, dietary diversity, food security, and educational status. Public health interventions targeting these identified factors are recommended to reduce the double burden of malnutrition.

A study in the Mt. Cameroon Health Region investigated micronutrient deficiencies and their effects on maternal hemoglobin levels. Maternal mean hemoglobin levels were significantly higher (*P* < 0.001) in women consuming heme iron (11.08 ± 1.35) and vitamin A food groups (11.34 ± 1.30) compared to those not consuming these groups (10.54 ± 1.19 and 10.74 ± 1.31, respectively). The findings emphasize the importance of increasing maternal nutritional awareness and counseling during the antenatal period to reduce the burden of anemia.

The study investigated the association between dietary diversity and the risk of cataracts using data from the Chinese Longitudinal Healthy Longevity Survey (CLHLS) spanning 2008 to 2018. Data from 47,395 participants with an average age of 86.1 years were analyzed, revealing that a higher overall dietary diversity (odds ratio = 0.74) and plant-based diets (odds ratio = 0.65) were associated with a reduced risk of cataracts. Conversely, an animal-based diet was shown to slightly increase the risk (odds ratio = 0.90). Incorporating a diverse diet may serve as a cost-effective intervention strategy for the prevention of cataracts.

These important findings underscore the necessity of implementing educational programs on the importance of dietary diversity to improve the nutritional status and health of women, achieve better birth outcomes, and also enhance the nutritional status of the elderly. Additionally, campaigns should be conducted within communities to promote healthy food choices and encourage the use of diverse ingredients. These strategies should be adapted to each local culture and available resources. By strengthening community partnerships and providing sustainable and practical solutions, we can improve the nutritional status of pregnant women and promote healthy birth outcomes. Additionally, as global aging progresses, it is crucial that public health policies and intervention programs aimed at improving the nutritional status of the elderly and extending healthy life expectancy are designed with consideration for local needs and conditions. This approach will enhance the efficiency and effectiveness of implementation. In other words, promoting dietary diversity goes beyond simply increasing the variety of foods consumed; it also entails ensuring access to nutrient-rich options. This requires addressing economic, cultural, and logistical barriers to healthy eating.

## Author contributions

MK: Writing – original draft, Writing – review & editing. FT: Writing – review & editing.
